# Thermo-Sensitive Genic Male Sterile Lines of Neo-Tetraploid Rice Developed through Gene Editing Technology Revealed High Levels of Hybrid Vigor

**DOI:** 10.3390/plants11111390

**Published:** 2022-05-24

**Authors:** Yang Chen, Muhammad Qasim Shahid, Jinwen Wu, Ruilian Deng, Zhixiong Chen, Lan Wang, Guoqiang Liu, Hai Zhou, Xiangdong Liu

**Affiliations:** 1 State Key Laboratory for Conservation and Utilization of Subtropical Agro-Bioresources, South China Agricultural University, Guangzhou 510642, China; cy530871943@163.com (Y.C.); qasim@scau.edu.cn (M.Q.S.); jwwu@scau.edu.cn (J.W.); rldeng@stu.scau.edu.cn (R.D.); chenzx@scau.edu.cn (Z.C.); wanglan@scau.edu.cn (L.W.); 984702957@stu.scau.edu.cn (G.L.); 2 Guangdong Laboratory for Lingnan Modern Agriculture, South China Agricultural University, Guangzhou 510642, China; 3 Guangdong Provincial Key Laboratory of Plant Molecular Breeding, South China Agricultural University, Guangzhou 510642, China; 4 College of Agriculture, South China Agricultural University, Guangzhou 510642, China; 5 College of Life Sciences, South China Agricultural University, Guangzhou 510642, China

**Keywords:** neo-tetraploid rice, CRISPR/Cas9, thermo-sensitive genic male sterile, *TMS5*, polyploid hybrid rice

## Abstract

Neo-tetraploid rice, which developed from the progenies of autotetraploid hybrid by our research group, is a useful germplasm with high fertility and strong heterosis when they crossed with other autotetraploid rice lines. The CRISPR/Cas9-mediated *TMS5* gene editing system has been widely used in diploid rice, but there are few reports in tetraploid rice. Here, we used CRISPR/Cas9 technology to edit the *TMS5* gene, which is a temperature sensitive gene controlling the fertility in diploid rice, in neo-tetraploid rice to develop male sterile lines. Two mutant lines, H2s and H3s, were developed from the gene editing and displayed characteristics of thermo-sensitive genic male sterility. The daily mean temperatures of 23 °C to 26 °C were found to be critical for sterility (restrictive temperature) in H2s and H3s under both controlled (growth chambers) and natural growing conditions (field). Cytological observation showed the anther dysplasia appeared later in H2s and H3s than that of the *TMS5* mutant of diploid rice (E285s) under the same conditions. Then these mutant lines, H2s and H3s, were crossed with tetraploid rice to generate F_1_ hybrids, which exhibited obvious advantages for effective number of panicles, total grains and seed setting. The high levels of hybrids heterosis were maintained for several generations that can save seed cost. Our research provides an effective way of developing thermo-sensitive genic male sterility (TGMS) lines of tetraploid rice using gene editing, which will accelerate the utilization of polyploid heterosis.

## 1. Introduction

Rice (*Oryza sativa* L.) is one of the most important food crops in the world, especially in Asia. Increasing grain yield is a long-run aim in crop breeding to increase the production and meet the requirement for global food security. However, rice yield is stagnant from the past few years. Therefore, it is very important for rice breeders to evolve new cultivars with high yield by using modern and conventional approaches. As a new germplasm resource, autotetraploid rice has the advantages of high yield potential, nutrition, strong resistance and heterosis [1,2,3,4,5,6,7]; however, low seed setting limits its commercial utilization [3,8,9,10,11,12,13,14,15,16]. In this case, it is of great significance to generate high-fertility tetraploid rice germplasm [4,15,16,17,18,19,20].

In order to solve the problem of low fertility of autotetraploid rice, our research team developed a new type of tetraploid rice (known as “neo-tetraploid rice”), including Huaduo 1 to Huaduo 5 and Huaduo 8 [4,21,22,23,24,25]. The neo-tetraploid rice showed normal fertility and strong yield heterosis when crossed with low fertility autotetraploid rice lines [4,23,24]. Comparative genomic analysis revealed polyploid rice as an independent group with unique classification and containing novel allele, such as *HSP101-1* [25]. At least ten genes were found to be associated with the fertility of neo-tetraploid rice [21,22,23]. Neo-tetraploid rice could become a very useful germplasm to promote the breeding of polyploid rice, especially for heterosis utilization.

Heterosis or hybrid vigor is commonly known as the superior performance of F_1_ hybrid compared to the parents. Asian cultivated rice is divided into two main subspecies i.e., *indica* and *japonica*, and their intersubspecific F_1_ hybrids showed high hybrid vigor for yield and other agronomic traits [26,27,28,29]. Hybrid rice is being cultivated from last four decades and occupies more than 50% of the total rice area in China [30]. The two-line hybrid rice method is developed by Chinese researchers to utilize rice heterosis using photoperiod-thermo-sensitive genic male sterile lines (PTGMS) [31]. Compared with three-line hybrid rice, two-line hybrid rice has several advantages, such as no need of maintainer line, a wide range of lines can be used as male parent that is an ideal way to utilize the heterosis between indica and japonica subspecies, and high combination probability of producing better grain quality and high yield, and it has become one of the main techniques for hybrid rice production in China [31,32]. At present, two-line hybrid rice accounts for about half of the area planted with hybrid rice.

The thermo-sensitive male sterile (TGMS) gene, *TMS5*, is the main genetic resource for the production of TGMS lines for two-line hybrid rice breeding [33]. It had shown that 24 of the 25 major PGMS and TGMS diploid rice lines in China contain *TMS5* gene, indicating that *TMS5* gene is the main sterile gene controlling thermo-sensitive genic male sterility in rice [33,34]. *TMS5* gene was cloned in two TGMS lines, An’nong s-1 and Zhu 1s, which revealed the molecular mechanism of thermo-sensitive male sterility in diploid rice [33].

The PTGMS of autotetraploid displayed obvious fertility conversion characteristics compared to diploid photo-and thermos-sensitive genic male sterile (PTGMS) line [35]. Two PTGMS of polyploid rice were evolved by the conventional breeding method, i.e., from the crossing generations of PTGMS of autotetraploid rice [35]. However, it takes many years to develop a new PTGMS of polyploid rice, i.e., the cycle of conventional breeding method is very long. CRISPR/Cas9 gene editing system is a powerful technology to create specific mutations, and had been used to knock out *TMS5* gene to develop new TGMS lines of diploid rice [34,36,37,38,39]. However, there is little known about the development of TGMS lines for two-line hybrid breeding of tetraploid rice, especially neo-tetraploid rice. Therefore, CRISPR/Cas9 technology was used to knock out *TMS5* gene of two neo-tetraploid rice lines, and two new polyploid rice TGMS lines were developed in the present study. At the same time, the pollen fertility and seed setting rate of *TMS5* mutants under different temperature treatments were recorded to determine the temperature for sterility-fertility transition. The cytological characteristics of anthers and pollen development in different ploidy rice under the same environment were observed. The promising results of the present study will accelerate the breeding procedure of tetraploid hybrid rice TGMS lines for the production of two-line polyploid hybrid rice.

## 2. Results

### 2.1. Development of TGMS Lines by the Editing of Thermos-Sensitive Genic Male Sterile 5 (TMS5) Locus in Neo-Tetraploid Rice

**Figure 1:**
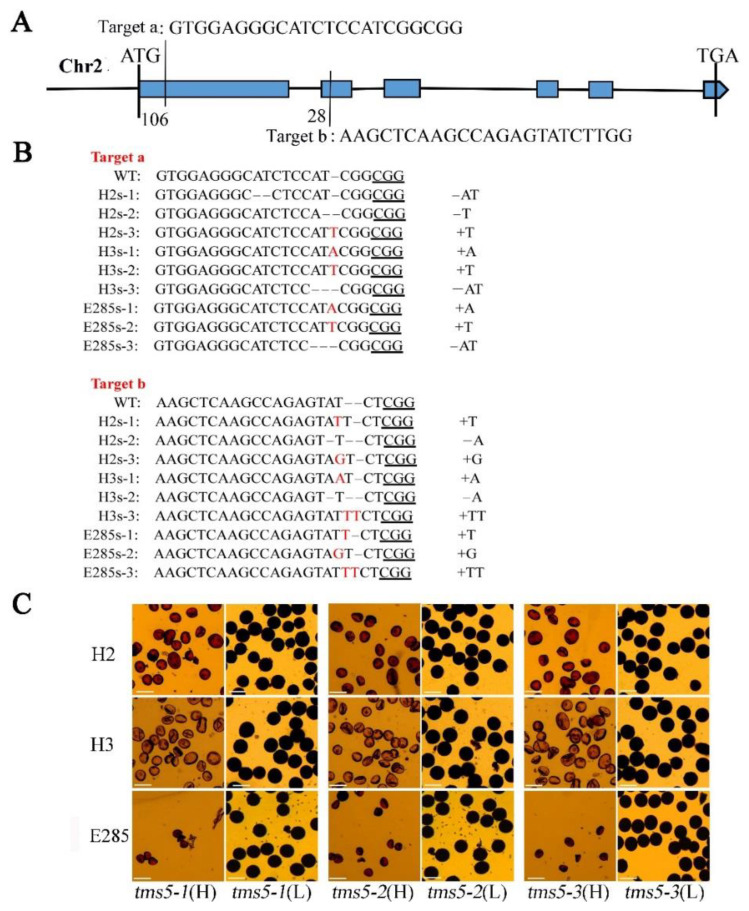
Target location of *TMS5* gene, homozygous mutation sequence of T1 generation and pollen fertility. (**A**) Structure of *TMS5* gene. The 20 bp adjacent to the underlined protospacer adjacent motifs (PAMs) in wild-type sequence are CRISPR-Cas9 target sequence. Six exons are represented by blue boxes. The first blue box and vertical line indicate 106–128 base position of the first exon that shows the location of the target TMS5a and second blue box and vertical line represent the 28–46 base position of the second exon that shows the location of the target b. (**B**) The target sites of *TMS5* gene are consistent with the mutant sequence. Inserted nucleotides displayed in red; (**C**) Pollen fertility of different mutant types at high and low temperatures. H, high temperature; L, low temperature; Bars = 50 μm.

To understand the performance of neo-tetraploid rice after the deletion/mutation of *TMS5*, the gene was edited by CRISPR/Cas9 editing system in two neo-tetraploid rice, H2 and H3. A diploid rice mutant, *TMS5*, was used as control (Figure 1). A total of 17, 16 and 6 T_0_ transgenic plants from the gene editing of H2, H3 and E285 were obtained, respectively. Three homozygous mutants were screened from T_1_ for each material, namely H2s-1, H2s-2, H2s-3, H3s-1, H3s-2, H3s-3, E285s-1, E285s-2 and E285s-3, and all these homozygous mutants were T-DNA free. There were different types of mutations in the “target a” of *TMS5* of the above materials, including insertion of 1 bp ‘T’ (thymine), 1 bp ‘A’ (adenine), deletion of 1 bp ‘T’, 1 bp ‘C’ (cytosine), 2 bp ‘AT’ and 4 bp ‘CCAT’. Moreover, different types of mutations such as insertion of 1 bp ‘T’, 1 bp ‘A’, 1 bp ‘G’ (guanine), 2 bp ‘TT’ and deletion of 1 bp ‘A’ occurred at the target site b of *TMS5* (Figure 1A,B). In this study, we selected the homozygous T_1_ plants to develop T_2_ plants.

Diploid rice.

**Figure 2:**
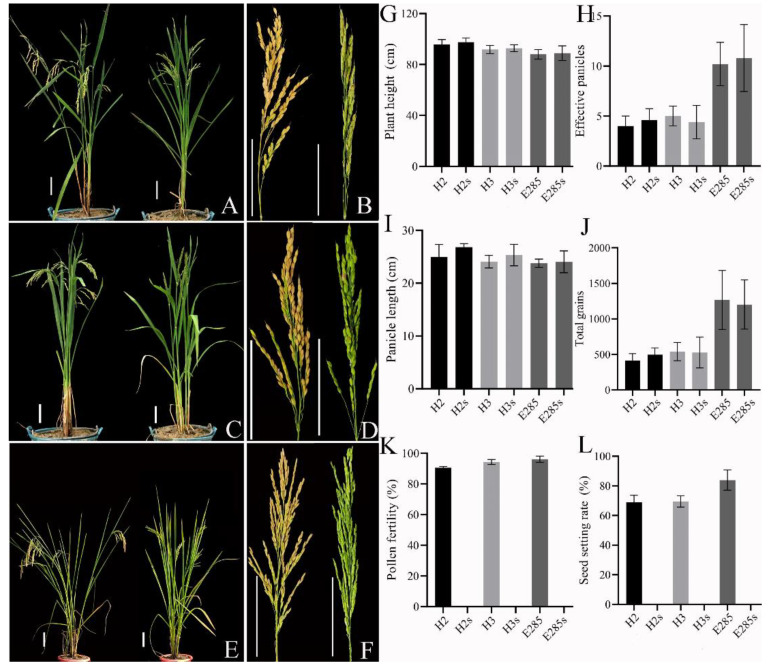
Identification of morphological and agronomic traits of *TMS5* mutants in the field. (**A**) The plant type of wild-type and *TMS5* mutant of H2. (**B**) Mature panicles of wild-type and *TMS5* mutant of H2. (**C**) The plant type of wild-type and *TMS5* mutant of H3. (**D**) Mature panicles of wild-type and *TMS5* mutant of H3. (**E**) The plant type of wild-type and *TMS5* mutant of E285. (**F**) Mature panicles of E285. Bars = 10 cm. (**G**) Plant height of WT and *TMS5* mutant. (**H**) Effective number of panicles of WT and *TMS5* mutant. (**I**) Panicle length of WT and *TMS5* mutant. (**J**) Total number of grains of WT and *TMS5* mutant. (**K**) Seed setting rate of WT and *TMS5* mutant. (**L**) Pollen fertility of WT and *TMS5* mutant; Five plants of WT and *TMS5* mutants were selected.

Both *TMS5* mutant and WT plants grown in the field showed similar morphological traits during different growth stages, while significant differences were detected in the pollen fertility and seed setting (Figure 2A–L). The pollen fertility and seed setting of H2 plants were 90.56% and 69.06%, and those of H3 were 94.43% and 69.49%, and 96.15% and 83.91% of E285, respectively (Figure 2K,L). The pollen fertility and seed setting of the *TMS5* mutants were 0 under high temperature (Figure 1C and Figure 2K,L), and different *TMS5* mutation types displayed the almost normal pollen fertility performance at low temperatures (Figure 1C). These results showed that *TMS5* mutation in tetraploid rice resulted in the thermo-sensitive sterility characteristics, including typical high-temperature sterility and low-temperature fertility, and these *TMS5* mutants of H2, H3 and E285 hereafter mentioned as H2s, H3s and E285s, respectively. Among them, H2s and H3s are new TGMS (thermo-sensitive male sterile) lines of neo-tetraploid rice, while E285s is a TGMS line of diploid rice.

### 2.2. Cytological Observation of Pollen Development in H3s and E285s

**Figure 3:**
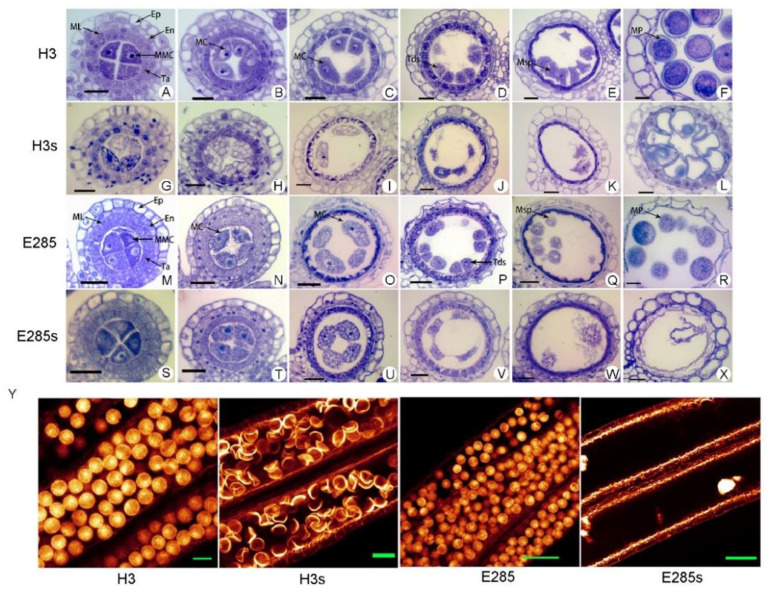
Comparison of transverse sections of the anthers of wild-type and *TMS5* mutants, H3s and E285s, grown at high temperature. (**A**–**F**) and (**M**–**R**) semi-thin sections of wild type anthers, (**G**–**L**) and (**S**–**X**) semi-thin sections of *TMS5* mutant anthers. (**A**,**G**,**M**,**S**) Microspore mother cell formation stage; (**B**,**H**,**N**,**T**,**C**,**I**,**O**,**U**) meiosis stage I; (**D**,**J**,**P**,**V**) tetrad stage; (**E**,**K**,**Q**,**W**) single microspore stage; (**F**,**L**,**R**,**X**) mature pollen stage. Ep, epidermis; En, endothecium; Ta, tapetum; MMC, microspore mother cell; MC, meiotic cell; Tds, tetrads; Msp, microspore parietal cell; MP, mature pollen. (**Y**) Anthers and mature pollen grains between the wild type and *TMS5* mutant using WE-CLSM analysis. Bars = 50 μm.

Comparative cytological observations of the pollen development of H3s, E285s and E285 (WT), which planted in the early season (high temperature condition), were carried out by semi-thin section technique. From pre-meiosis to tetrad stage, the pollen mother cell abnormalities of H3s and E285s were roughly the same, but the differences appeared from single microspore stage. At the pre-meiosis stage, the pollen mother cells of H3s and E285s began to vacuolate, while these cells were found normal in E285 (Figure 3A,M). At meiosis stage, microcytes were less in number and contain large amounts of small vacuoles in H3s and E285s compared to E285 (Figure 3B,C,H,I,N,O,T,U). At the tetrad stage, microcytes of the two H3s experienced more remarkable increase in vacuolization and intercellular adhesion (Figure 3D,J,P,V). At the single microspore stage, only a few microspores were observed in H3s, however, the pollen cells of E285s formed large protoplasts, but (Figure 3E,K,Q,W). At mature pollen stage, the pollen grains of E285 (wild-type) were full of starch and tapetum degenerated completely. However, pollen cells showed typical abortive phenotype in H3s (Figure 3F,L). Interestingly, no pollen grains were observed in the anther locule of E285s. Moreover, whole-mount eosin B-staining confocal laser scanning microscopy (WE-CLSM) observations of anthers verified that pollen development was significantly abnormal in H3s compared to its wild type H3 (Figure 3Y).

A comprehensive comparative study was carried out on two aspects by WE-CLSM observation of pollen development and the observation of anther semi-thin section. The results showed that the anther development processes of E285 and H3 were consistent. The anther development of E285s and H3s experienced vacuolar programmed death, and finally showed pollen sterility. The epidermis, endothelium and mesoderm of the mutants developed normally and tapetum degraded slightly earlier. Under the same conditions, the vacuolation time of *TMS5* mutants of H3 was later than that of E285 during pollen development. Finally, the pollens of *TMS5* mutants of E285s were completely degraded at start of meiotic stage (Figure 3Y), while pollens of *TMS5* mutants of H3s displayed abortion at single microspore stage (Figure 3Y).

### 2.3. Critical Sterility Temperature for the TGMS Lines of Neo-Tetraploid Rice

**Figure 4:**
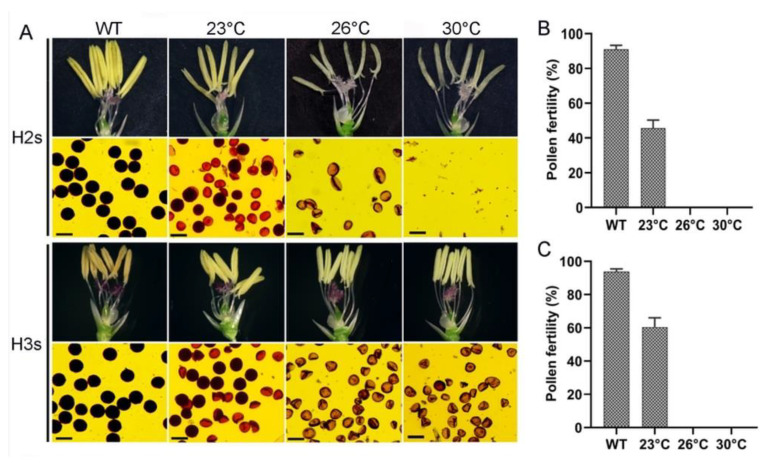
The fertility performance of TGMS lines at different temperatures. H2s and H3s were selected from the *TMS5* mutants of H2 and H3 lines, respectively. (**A**) Pollen fertility of TGMS lines at different temperatures. H2s and H3s were grown at 23 °C, 26 °C and 30 °C, with light conditions of 13.5 h and dark conditions of 10.5 h. (**B**) Pollen fertility of H2s line at different temperatures. (**C**) Pollen fertility of H3s line at different temperatures.

In order to exploit novel TGMS lines for heterosis breeding, it is very important to determine the critical sterility-inducing temperature (i.e., to minimize fertility restoration or self-fertility). As a result, two experiments were carried out in the present study. The TGMS lines of neo-tetraploid rice, H2s and H3s, were grown in growth chambers with daily average temperatures of 23 °C, 26 °C and 30 °C. At high temperatures (26 °C and 30 °C), the anthers of two TGMS lines were white and thin, and the pollen grains displayed irregular shapes, such as triangular or prismatic. At low temperature (23 °C), the anthers showed yellow and near normal size, similar to those of wild type (H2 and H3), and high percentage of normal pollens were observed (Figure 4A). At 23 °C, the pollen fertility and seed setting of H2s were 45.73% and 21.04%, and those of H3s were 60.46% and 32.22%, respectively (Figure 4B,C; Table S1).

In other field experiments, anther and pollen showed significant differences during different natural growth stages. From June to October, H2s displayed pollen sterility under natural conditions in Guangzhou, and pollen fertility of H2s restored in early November (Table S1). Similarly, pollens of H3s were completely sterile from June to October in Guangzhou and resumed fertility in early November with an average of 43.77% pollen fertility (Table S2). The above results revealed that H2s and H3s can be used for hybrid seed production during June to October, and reproducing seeds during November to early December in Guangzhou based on their growth habit and fertility restoration.

### 2.4. Heterosis Analysis of TGMS Lines of Neo-Tetraploid Rice Crossed with Autotetraploid Rice

A total of 15 F_1_ hybrids were generated by TGMS lines of neo-tetraploid rice by crossing with different autotetraploid rice lines. The main agronomic and yield related traits of these F_1_ hybrids improved significantly compared to their corresponding parents, especially the traits such as plant height, effective panicle number, total grain number, filled grain number and yield per plant (Table 1). The F_1_ hybrids generated by H2s produced plant height higher than 97.80cm, number of effective panicles greater than 6.60, the number of total grains higher than 820.40, the number of filled grains more than 536 and the yield per plant larger than 17.10 g. Similarly, plant height was higher than 97.50 cm, number of effective panicles was more than 7, total grains were more than 1029.33, filled grains were more than 787, and yield per plant was higher than 27.37g in the F_1_ hybrids generated by crossing with H3s (Table 1). Super-parental heterosis was also conducted in this study and found nine agronomic traits of all F_1_ hybrids showed positive mean super-parental heterosis, including number of effective panicles (79.07%), number of total grains (166.41%), number of filled grains (547.23%), seed setting rate (115.19%) and yield per plant (554.51%) (Table S3). The highest high-parent heterosis was detected in the combinations of T423 × H3s and T437 × H2s (Table S3), and seven main agronomic traits exhibited positive average competitive advantages than its higher parent.

Moreover, we observed the data of important agronomic traits up to F_3_ generation. The yield traits of most of the combinations showed slightly downward trend in F_2_ and F_3_ generations compared to the previous generation, but no obvious segregation was observed. Interestingly, F_3_ generation of T485 × H3s showed an increase in economic yield and harvest index compared to the previous generations (Table 2). These results show that tetraploid hybrid rice can be crossed once and grown for multiple generations, significantly reducing the cost of hybrid seed production.

## 3. Discussion

### 3.1. Gene Editing through CRISPR/Cas9 System Provides an Effective Way of Developing TGMS Lines of Tetraploid Rice

Generally, it takes 7 to 8 years to develop a new TGMS line of rice using conventional breeding method. While, it needs only 1 to 2 years to generate a new line using CRISPR/Cas9 gene editing system, which could accelerate the breeding process of TGMS lines for two-line hybrid breeding. CRISPR/Cas9 gene editing technique has been widely used to create new TGMS lines of rice, and about 20 lines have been reported in diploid rice [34,36,37,38,39], for example, CRISPR/Cas9 gene editing technique was employed to create *TMS5* mutants, and 11 new TGMS lines were developed [34]. *TMS5* mutants in rice were generated based on CRISPR/Cas9 technology, which showed that the fertility transition temperature was 28 °C [36]. A TGMS line, YK17S, was developed from *indica* rice cultivar, Zhongjiazao17, by CRISPR/Cas9, whose sterility transition temperatures was 24 and 26 °C [39], while another TGMS rice line, GH89, was created by CRISPR/Cas9 with critical sterility-inducing temperature of about 24 °C [37]. Recently, six lines of *japonica* and two *indica* TGMS lines were developed by using CRISPR/Cas9 gene editing technology, and the critical sterility-inducing temperatures were between 28 °C and 32 °C, and 23.5 °C to 28 °C, respectively [38]. All the above studies revealed that CRISPR/Cas9 gene editing system could be applied successfully to generate TGMS lines of diploid rice, however, little is known about its application in polyploid rice. Therefore, CRISPR/Cas9 technology was employed to knock out *TMS5* in neo-tetraploid rice, and two TGMS lines were developed that showed sterility transition at 23 to 26 °C in this study, which was nearly consistent with TGMS lines PS006 (23.6 °C) and PS012 (24.4 °C) produced by conventional breeding method [35]. The two TGMS lines of neo-tetraploid rice displayed similar morphological traits compared to wild type, except for the sterility at high temperature and fertility at low temperature, which demonstrated that it is feasible to create new TGMS lines by gene editing technology in polyploid rice.

### 3.2. Pollen Sterility Is Different between the TGMS Lines of Neo-Tetraploid and Diploid Rice

In this study, the cytological observations of pollen abortion were conducted in diploid and tetraploid *TMS5* mutants under same conditions, and we found some interesting results. The pollen abortion started at the pollen mother cell stage in diploid rice and completely degraded at the meiosis stage, which was consistent with the previous study [34]. Anther abortion originated from cytoplasmic vacuolization in the late pollen mother cell stage in An’nong s-1, a TGMS line, and its abortion characteristics were also the same as intercellular adhesion and disintegration, and then the loss of cell structure and eventually disappeared. At 30 °C, the pollens of An’nong s-1 degraded and completely disappeared during meiosis, while pollen abortion occurred at 26 °C [33]. Here, the formation of vacuoles (vacuolation) started from the pollen mother cell stage in *TMS5* mutants of diploid rice and vacuolation was the most frequent during meiosis, and large protoplasts were observed at single microspore stage. Finally, pollens of diploid *TMS5* mutant degraded and no mature pollen was found. Under the same growing conditions, the vacuolation began from the pollen mother cell stage in tetraploid *TMS5* mutants, and few microspores were observed at single microspore stage. Finally, typical abortive pollens were detected in the tetraploid mutants. Cytological studies of Pei’ai 64s, a PGMS line, showed two types of abnormal pollen development [40]. In our work, as mentioned above, the cytoplasm of microspore mother cells was abnormal at the early stage of meiosis in diploid mutants, and gradually vacuolated and eventually disintegrated during the subsequent development. In contrast, after the early formation of microspores, all the outer walls of microspores were abnormal, and it was difficult to distinguish between the boundary of surface layer and inner layer, which resulted in the failure of inner wall formation, and yielded typical abortive pollens in neo-tetraploid rice. *UbL40* gene is induced by high temperature during the regulation mode of *TMS5* gene function and specifically expressed in pollen mother cells [33,41,42].

### 3.3. Hybrids Generated by TGMS Lines of Neo-Tetraploid Rice Displayed Obvious Heterosis and Maintained for Several Generations

Heterosis or hybrid vigor is an important phenomenon and has been applied successfully to increase the yield of various plant species. Polyploid rice hybrids displayed strong heterosis in yield-related traits and could be a new way for rice breeding [4,16]. Three-line hybrid breeding system was established to utilize autotetraploid rice heterosis, and about 6 male sterile lines, more than 20 maintainer lines and more than 20 restorer lines of autotetraploid rice were developed, including an excellent combination, T462A × T4509 [6]. The hybrid of neo-tetraploid rice crossing with autotetraploid rice not only exhibited biological advantages, but also showed obvious yield advantage. Our research group has used neo-tetraploid rice to cross with different types of tetraploid rice lines and prepared more than 70 combinations in the last five years. We observed significant F_1_ heterosis, including mid parent heterosis and super parent heterosis for yield related traits, such as the number of grains per plant, seed setting and yield per plant, which reached a significant level. According to the statistics of 53 combinations, the average heterosis of mid parent for number of seeds, seed setting and yield per plant were 84.41%, 32.94% and 106.58%, respectively. The average value of super parent dominance was 59.27%, 13.83% and 75.82%, respectively [4,22]. In the present study, two TGMS lines of neo-tetraploid rice were generated by CRISPR/Cas9 gene editing technology and were used to cross with different tetraploid rice lines to analyze heterosis. The results showed competitive advantage for most of the traits in different generations of several hybrids, and it was the most obvious in F_1_ generation. Interestingly, we did not notice any obvious segregation in F_2_ and F_3_ generation of tetraploid rice, which showed that the utilization of tetraploid heterosis is feasible even in further generations. In conclusion, TGMS lines generated in this study exhibited a promising application for the development of two-line hybrid breeding system for tetraploid rice with high fertility that can be maintained for several generations.

## 4. Materials and Methods

### 4.1. Plant Materials and Growing Conditions

Two neo-tetraploid rice lines, Huaduo 2 (H2) and Huaduo 3 (H3) that were developed by our research group, and one diploid rice (Huanghuazhan, E285, control) were used as receptor materials for genetic transformation. The CRISPR/Cas9 gene editing vector of *TMS5* was designed and constructed according to the steps as follow: 19 to 20 bases upstream of PAM motif were selected as the candidate target sequence. There are six exons in rice thermo-sensitive sterile gene (*TMS5*). The sgRNA binding targets were set at the 106–128 base position of the first exon and the 28–46 base position of the second exon [34]. The CRISPR/Cas9 gene editing vector was sent to Wuhan Bo’yuan Company for genetic transformation.

All materials, including putative transgenic gene edited plants and their wild types, were grown at the farm of South China Agricultural University (Guangzhou: 23_N, 113_E, Guangdong), and managed according to the recommended protocol for the area. In order to test the effect of temperature on pollen development, T_2_ mutants and their parents/wild type materials (CK) were planted in the growth chambers with daily mean temperatures of 23 °C, 26 °C or 30 °C and a 13.5h photoperiod. Moreover, the mutants and wild type (H2 and H3) were planted in different growing seasons at the same farm, including March, June and August, to determine the pollen fertility restoration period in Guangzhou for the collection of seeds.

### 4.2. Mutation-Sites Detection of Transgenic Plants Generated by CRISPR/Cas9 Gene Editing of TMS5

The target fragment, extracted from the leaves by CTAB method, was amplified by DNA-binding specific primers, and the sequencing results of the target fragment amplified by sequencing primers were used as control. The positive transgenic plants were identified by PCR amplification of hygromycin gene in the T_1_ generation. The genotype of *TMS5* was identified by sequencing results, and homozygous mutant plants were obtained. The T_2_ generation was obtained by self-pollination for identification and fertility observation. The relevant PCR primers for these steps are listed in Table S4.

### 4.3. Cytological Observations of Pollen Fertility and Anther Development of tms5 Mutants and Wild Types

The young panicles of *TMS5* mutants and their wild-types (H2 and H3) were fixed in FAA solution (50% ethanol: acetic acid: methyl aldehyde = 89:6:5) for 48 h, then washed three times with 70% ethanol solution, and their anthers were taken by dissection for semi-thin section. The anthers were dehydrated serially in 80%, 90% and 95% ethanol solution for 30 min, respectively. Then Leica 7022 historesin embedding kit was used for embedding and polymerization at room temperature. The cross section of anther with 3 μm thickness was cut by Leica RM2235 rotary microtome, stained with 1% toluidine blue, and observed and photographed under microscope (Motic BA 200, Motic, Xiamen, China). The whole mount eosin B confocal laser scanning microscopy (WE-CLSM) was also employed to observe the development of embryo sacs in TMS5 mutant and its wild-type according to Ghouri et al. [43] with minor modifications. The samples were sequentially hydrated in 70%, 50%, 30%, 10% ethanol and ddH_2_O for 15 min each. Then the anthers were stained with eosin B staining for 1–2 h. After that, they were dehydrated in 10%, 30%, 50%, 70%, 90% and 100% ethanol (three times) for 30 min. Finally, the absolute ethanol was changed into a mixture solution (ethanol and methyl salicylate = 1:1) for 1h, and then to pure methyl salicylate for 1h. The samples were scanned under a laser scanning confocal microscope (Leica SPE).

### 4.4. Analysis of Agronomic Traits in the Hybrids of TGMS Lines of Neo-Tetraploid Rice Crossed with Autotetraploid Rice

In order to evaluate the heterosis of the hybrids of TGMS lines of neo-tetraploid rice crossed with autotetraploid rice, a total of 20 F_1_ hybrids were generated by crossing tetraploid rice lines as male parent with TGMS lines of neo-tetraploid rice. Moreover, F_2_ and F_3_ generations of all hybrids were developed by self-crossing of F_1_ or F_2_ population. F_1_ hybrids were planted at the farm of South China Agricultural University in 2019, while F_2_ and F_3_ generations were grown in 2020. Agronomic traits, including plant height, panicle length, effective panicle number, total grain number, filled grain number, seed setting rate, yield per plant and 1000-grain weight were investigated according to Guo et al. [4]. The yield concerned traits, including economic yield, biological yield and harvest index, were calculated in F_2_ and F_3_ generations of all combinations. Economic output refers to grain output per unit land area and harvest index is the ratio of economic output to biological output.

## 5. Conclusions

We employed CRISPR/Cas9 technology to develop thermo-sensitive genic male sterility (TGMS) lines of neo-tetraploid rice. The daily mean temperatures of 23 °C to 26 °C were found to be critical for pollen sterility (restrictive temperature) in both *TMS5* mutants of neo-tetraploid rice. Whole-mount eosin B-staining confocal laser scanning microscopy (WE-CLSM) observations of anthers verified that pollen development was significantly abnormal in *TMS5* mutant compared to wild type, and anther dysplasia appeared later in *TMS5* mutants than *TMS5* mutant of diploid rice (E285s) under the same conditions. Then these *TMS5* mutant lines were crossed with tetraploid rice to generate F_1_ hybrids, which exhibited obvious advantages for yield and yield-related traits, and high heterosis was maintained for several generations. These *TMS5* mutants could be used for the two-line hybrid rice breeding that would accelerate the utilization of polyploid heterosis.

## Figures and Tables

**Table 1:** Grain yield and yield components of wild type, parents and F_1_ hybrid.

Material Name	PH (cm)	PL (cm)	EP	TGP	FGP	SS (%)	GYP (g)	KGW(g)
H2	94.67 ± 4.16	24.98 ± 2.33	4.40 ± 1.14	413.40 ± 97.23	285.20 ± 68.98	69.06 ± 4.58	10.68 ± 2.47	37.54 ± 2.52
H2s × T437	101.33 ± 5.91	28.33 ± 1.65	13.33 ± 3.68	1546.00 ± 305.15	1180.00 ± 192.17	77.02 ± 4.36	39.60 ± 7.23	33.81 ± 4.30
T437	94.60 ± 8.71	24.72 ± 0.73	5.00 ± 1.58	332.80 ± 108.98	65.00 ± 30.49	19.00 ± 5.84	2.83 ± 1.54	43.09 ± 6.82
H2s × T445	97.80 ± 3.11	26.60 ± 0.98	6.60 ± 1.14	820.40 ± 264.19	536.00 ± 195.01	65.10 ± 7.67	17.10 ± 5.65	32.27 ± 1.93
T445	90.40 ± 4.04	26.00 ± 1.80	5.20 ± 1.92	670.20 ± 229.73	297.20 ± 145.92	42.31 ± 7.99	10.21 ± 5.10	33.93 ± 4.47
H2s × T473	104.33 ± 2.87	31.57 ± 0.42	9.67 ± 2.49	1042.67 ± 119.67	769.00 ± 113.39	73.46 ± 2.53	27.23 ± 4.67	35.32 ± 1.31
T473	97.80 ± 3.19	25.52 ± 1.82	5.20 ± 1.64	566.60 ± 166.95	239.60 ± 49.94	44.27 ± 9.95	6.91 ± 1.22	29.04 ± 2.21
H3	94.40 ± 1.14	24.08 ± 1.19	6.00 ± 1.22	541.20 ± 128.30	373.60 ± 78.64	69.49 ± 3.79	11.62 ± 2.53	31.19 ± 2.75
H3s × T423	105.00 ± 2.35	23.92 ± 0.74	7.00 ± 2.00	1116.40 ± 421.80	840.40 ± 394.50	72.14 ± 17.07	31.11 ± 14.83	36.90 ± 0.55
T423	86.00 ± 3.39	16.62 ± 0.33	5.60 ± 1.52	294.20 ± 51.22	51.80 ± 28.24	16.82 ± 0.92	1.42 ± 0.85	26.88 ± 4.50
H3s × T424	97.00 ± 2.30	26.00 ± 2.72	9.00 ± 1.52	1429.00 ± 122.32	968.00 ± 59.72	67.74 ± 1.02	38.19 ± 3.54	39.45 ± 3.57
T424	77.00 ± 7.87	23.14 ± 0.77	2.20 ± 0.45	176.40 ± 26.08	16.20 ± 6.76	9.08 ± 3.39	0.51 ± 0.23	31.85 ± 2.59
H3s × T445	103.16 ± 3.86	28.22 ± 1.92	8.52 ± 2.11	1384.76 ± 797.14	1089.64 ± 691.34	77.45 ± 6.45	35.70 ± 19.83	33.58 ± 2.55
T445	90.40 ± 4.04	26.00 ± 1.80	5.20 ± 1.92	670.20 ± 229.73	297.20 ± 145.92	42.31 ± 7.99	10.21 ± 5.10	33.93 ± 4.47
H3s × T473	109.20 ± 4.87	30.76 ± 0.23	7.40 ± 1.52	1278.00 ± 421.72	893.60 ± 261.40	70.97 ± 7.92	32.09 ± 9.09	36.05 ± 1.70
T473	97.80 ± 3.19	25.52 ± 1.82	5.20 ± 1.64	566.60 ± 166.95	239.60 ± 49.94	44.27 ± 9.95	6.91 ± 1.22	29.04 ± 2.21
H3s × T485	107.76 ± 7.71	29.66 ± 0.92	9.20 ± 2.39	1241.24 ± 328.23	876.16 ± 242.68	69.95 ± 2.34	27.76 ± 6.86	32.01 ± 1.47
T485	82.60 ± 1.52	27.94 ± 1.18	6.40 ± 1.14	498.40 ± 88.17	294.20 ± 66.86	58.64 ± 4.81	9.88 ± 2.37	33.47 ± 1.33

Note: PH, plant height; PL, panicle length; EP, effective number of panicles per plant; TGP, total grains per plant; FGP, filled grains per plant; SS, seed set; GYP, grain yield per plant; KGW, 1000-grain weight.

**Table 2:** Yield performance of F_2_ and F_3_ hybrids.

Material Name	Biological Yield (Kg)	Economic Yield (Kg)	Harvest Index (%)
H2s × T437 (F_2_)	6.93 ± 2.01	1.28 ± 0.12	19.12 ± 3.39
H2s × T437 (F_3_)	7.07 ± 1.79	1.29 ± 0.19	18.54 ± 2.03
H2s × T445 (F_2_)	5.90 ± 1.21	1.62 ± 0.25	27.79 ± 2.84
H2s × T445 (F_3_)	5.99 ± 1.22	1.53 ± 0.19	25.85 ± 1.97
H2s × T473 (F_2_)	7.20 ± 0.69	1.55 ± 0.20	21.55 ± 1.77
H2s × T473 (F_3_)	7.75 ± 1.01	1.45 ± 0.03	18.96 ± 2.75
H3s × T423 (F_2_)	7.42 ± 0.99	1.25 ± 0.23	16.90 ± 2.30
H3s × T423 (F_3_)	5.82 ± 0.38	0.96 ± 0.05	16.43 ± 0.74
H3s × T424 (F_2_)	7.58 ± 1.28	1.42 ± 0.43	18.73 ± 5.54
H3s × T424 (F_3_)	5.82 ± 0.38	0.96 ± 0.05	16.43 ± 0.74
H3s × T445 (F_2_)	7.35 ± 1.00	1.68 ± 0.16	23.02 ± 1.66
H3s × T445 (F_3_)	6.99 ± 1.38	1.55 ± 0.18	22.56 ± 3.97
H3s × T473 (F_2_)	8.62 ± 0.68	1.51 ± 0.12	17.59 ± 0.88
H3s × T473 (F_3_)	6.59 ± 1.05 *	1.15 ± 0.15 *	17.51 ± 0.60
H3s × T485 (F_2_)	6.82 ± 1.51	1.83 ± 0.30	27.14 ± 2.33
H3s × T485 (F_3_)	6.47 ± 1.02	2.34 ± 0.13	36.60 ± 4.08 **

*, ** indicate significant differences at *p* < 0.05 and *p* < 0.01, respectively.

## Data Availability

All data have been included in the main text.

## Supplementary Materials

The following supporting information can be downloaded at: https://www.mdpi.com/article/10.3390/plants11111390/s1, Table S1: Primer sequences used in this study; Table S2: Pollen fertility and seed setting of self-crossed TGMS lines at different temperatures; Table S3: The fertility and heading date of H2s and H3s mutants under different sowing dates; Table S4: Heterosis analysis of F_1_ hybrid compared to the paternal.
