# Supplementary material for: Thermo-Sensitive Genic Male Sterile Lines of Neo-Tetraploid Rice Developed through Gene Editing Technology Revealed High Levels of Hybrid Vigor
Source: Plants (Basel). 2022 May 24;11(11):1390. doi: 10.3390/plants11111390 (PMC9182671; doi:10.3390/plants11111390)
Supplement: Supplementary file 1 [file plants-11-01390-s001.zip › plants-1704477-supplementary.pdf]

Additional files

Additional file 1. **Table S1** Primer sequences used in this study. **Table S2** Pollen fertility and seed setting of self-crossed TGMS lines at different temperatures. **Table S3** The fertility and heading date of H2s and H3s mutants under different sowing dates. **Table S4** Heterosis analysis of F_1 hybrid compared to the paternal

Table S1 Primer sequences used in this study

Primer name	Primer sequence (5'-3')
U-F	CTCCGTTTTACCTGTGGAATCG
gRNA-R	CGGAGGAAAATTCCATCCAC
Pps-GGL	TTCAGAGGTCTCTCTCGCACTGGAATCGGCAGCAAAGG
Pgs-GG2	AGCGTGGGTCTCGTCAGGGTCCATCCACTCCAAGCTC
Pps-GG2	TTCAGAGGTCTCTCTGACACTGGAATCGGCAGCAAAGG
Pgs-GGR	AGCGTGGGTCTCGACCGGGTCCATCCACTCCAAGCTC
TMS5a-F	GCCGTGGAGGGCATCTCCATCGG
TMS5a-R	AAACCCGATGGAGATGCCCTCCA
TMS5b-F	GGCAAGCTCAAGCCAGAGTATCT
TMS5b-R	AAACAGATACTCTGGCTTGAGCT
HPT-F	GTCCGTCAGGACATTGTTGGAG
HPT-R	GTCTCCGACCTGATGCAGCTCCGG

Table S2 Pollen fertility and seed setting of self-crossed TGMS lines at different temperatures

Average temperature	Photoperiod	H2s		H3s	
		Pollen fertility (%)	Seed setting (%)	Pollen fertility (%)	Seed setting (%)
23°C	13.5h	45.73±4.53	21.04±2.75	60.46±5.54	32.22±3.89
26°C	13.5h	0	0	0	0
30°C	13.5h	0	0	0	0

Table S3 The fertility and heading date of H2s and H3s mutants under different sowing dates.

Materials	Sowing date (Y–M–D)	Heading date (Y-M-D)	Pollen abortion Type	Pollen fertility (%)
H2s	2019-02-27	19-06-11	typical	0
	2019-03-07	19-06-17	typical	0
	2019-03-17	19-06-24	typical	0
	2019-03-27	19-06-27	typical	0
	2019-04-07	19-06-30	typical	0
	2019-04-17	19-07-10	typical	0
	2019-04-27	19-07-19	typical	0
	2019-05-07	19-07-28	typical	0
	2019-07-21	19-10-08	typical	0
	2019-08-02	19-10-18	typical	0
	2019-08-14	19-11-08	typical	0
		19-11-13	typical	29.42±11.51
		19-11-16	typical	49.64±9.53
	2020-08-05	20-10-27	typical	0
	2020-08-10	20-11-07	typical	15.93±8.13
		20-11-11	typical	81.16±7.54
H3s	2019-02-24	19-06-08	typical	0
	2019-07-20	19-10-13	typical	0
	2020-03-01	20-06-12	typical	0
	2020-08-05	20-11-03	typical	43.77±12.48
	2020-08-10	20-11-07	typical	72.13±8.62
		20-11-11	typical	62.94±6.59

Table S4 Heterosis analysis of F_1 hybrid compared to the paternal

Hybrid cross	Plant height (%)	Panicle length (%)	Effective panicle number (%)	Total grains (%)	Filled grains (%)	Seed set (%)	Grain length (%)	Grain width (%)	Yield per plant (%)	1000 grain weight (%)
H2s X T437	7.11	14.60	166.60	364.54	1715.38	305.37	-3.86	-8.87	1299.29	-21.54
H2s X T443	4.52	42.72	142.86	336.41	807.04	111.69	0.56	2.56	902.32	9.61
H2s X T442	5.51	6.90	138.64	148.10	322.45	82.15	-7.53	8.55	377.36	16.83
H2s X T445	8.19	2.31	26.92	22.41	80.35	53.86	-3.48	3.76	67.48	-4.89
H2s X T473	6.68	23.71	85.96	84.02	220.95	65.94	-5.70	-1.32	294.07	21.63
H3s X 25-26	-2.03	-5.04	18.18	74.00	166.10	50.80	-13.54	-2.82	151.74	-3.93
H3s X T423	22.09	43.92	25.00	279.47	1522.39	328.97	-2.28	10.05	2084.97	37.29
H3s X T437	11.52	16.30	70.00	280.11	1210.77	265.15	-1.16	-6.45	1027.47	-11.78
H3s X T449	21.30	4.64	20.83	35.93	109.71	48.67	-4.03	9.14	128.57	11.79
H3s X T442	4.04	6.27	115.91	134.13	265.60	57.26	-6.93	6.64	327.23	16.55
H3s X T445	14.12	8.52	63.85	106.62	266.64	83.07	-8.57	6.50	249.52	-1.02
H3s X T465	21.11	3.60	13.64	90.60	175.02	45.73	-13.50	3.13	173.40	-0.90
H3s X T473	11.66	20.53	42.31	125.56	272.95	60.31	-12.86	2.97	364.50	24.14
H3s X T485	30.46	6.16	43.75	149.04	197.81	19.29	-15.05	1.80	180.87	-4.37
